# Takotsubo syndrome: cognitive behavioural therapy, physical training, and brain function recovery in the BREAKOUT trial

**DOI:** 10.1093/eurheartj/ehaf441

**Published:** 2025-08-07

**Authors:** Hilal Khan, David T Gamble, Amelia Rudd, Ciprian Dospinescu, Calum Creaney, Graham Horgan, Andrea Holme, Heather M Wilson, David E Newby, Stuart R Gray, Rajeev Krishnadas, Christopher Williams, Gordon Waiter, Dana K Dawson

**Affiliations:** Aberdeen Cardiovascular and Diabetes Centre, University of Aberdeen, Polwarth Building, Foresterhill, Aberdeen, UK; Aberdeen Cardiovascular and Diabetes Centre, University of Aberdeen, Polwarth Building, Foresterhill, Aberdeen, UK; Aberdeen Cardiovascular and Diabetes Centre, University of Aberdeen, Polwarth Building, Foresterhill, Aberdeen, UK; Aberdeen Cardiovascular and Diabetes Centre, University of Aberdeen, Polwarth Building, Foresterhill, Aberdeen, UK; Aberdeen Cardiovascular and Diabetes Centre, University of Aberdeen, Polwarth Building, Foresterhill, Aberdeen, UK; Biomathematics & Statistics Scotland, Aberdeen, UK; Aberdeen Cardiovascular and Diabetes Centre, University of Aberdeen, Polwarth Building, Foresterhill, Aberdeen, UK; Aberdeen Cardiovascular and Diabetes Centre, University of Aberdeen, Polwarth Building, Foresterhill, Aberdeen, UK; University of Edinburgh, Edinburgh, UK; University of Glasgow, Glasgow, UK; Department of Psychiatry, University of Cambridge, Cambridge, UK; University of Glasgow, Glasgow, UK; Five Areas Ltd, Glasgow, UK; Aberdeen Cardiovascular and Diabetes Centre, University of Aberdeen, Polwarth Building, Foresterhill, Aberdeen, UK; Aberdeen Cardiovascular and Diabetes Centre, University of Aberdeen, Polwarth Building, Foresterhill, Aberdeen, UK

**Keywords:** Takotsubo syndrome, Brain functional magnetic resonance imaging, Exercise, Cognitive behavioural therapy, Behavioural modification

## Introduction

Takotsubo syndrome is an acute disease at the interface between the brain and heart, whereby a stressful trigger results in severe decline of cardiac function.^1^ We have previously shown that acute takotsubo syndrome is accompanied by a strong myocardial and systemic inflammatory process.^2^ We and others have demonstrated structural and functional brain differences between patients with acute takotsubo syndrome and age- and sex-matched control subjects.^3–7^ We hypothesized that behavioural modifications such as physical exercise training or cognitive behavioural therapy will improve structural and functional brain recovery patterns (reflected by changes in hippocampal and other brain volumes as well as in functional connectivity) after acute takotsubo cardiomyopathy, relative to current standard care.

## Methods

### Study design and population

An open-label randomized controlled trial approved by the North of Scotland Research Ethics Committee (20/SC/0305) was conducted between October 2020 and July 2023. Patients were invited to participate if they met contemporaneous diagnostic criteria for acute takotsubo syndrome^8^ and gave written informed consent.

### Study assessments and randomization

Baseline assessments were performed within 3 weeks of the hospitalized index event and follow-up after 12 weeks of study intervention. Participants were randomized 1:1:1 into one of three arms: (i) standard care, (ii) physical exercise training, or (iii) cognitive behavioural therapy.

### Study interventions

*Physical exercise training* was delivered as a 12-week personalized training regime targeted to heart rate reserve or the Borg rating perceived exertion scale if they were on beta-blocker.

*Cognitive behavioural therapy* was delivered as a series of 12 life skills sessions, purposefully adapted for a takotsubo syndrome sufferer from the original Living Life to the Full classes.

*Standard care* was local to each centre and did not include behavioural modification.

### Study assessments

*Brain magnetic resonance imaging protocol and analysis* methods have previously been described in detail.^5^ All scans were performed at 3T (Philips Healthcare, Best, Netherlands) with a 32-channel phased-array coil and analysis blinded to group allocations performed on FreeSurfer v7.1.1, Functional Connectivity and Diffusion Toolbox, respectively.

*Psychology, social function, and symptoms questionnaires* Hospital Anxiety and Depression Scale and Perceived Stress Scale were completed.

*Salivary cortisol* on awakening was batch-analysed using a liquid chromatography and tandem mass spectrometer on Waters Acquity UPLC system coupled to a XEVO TQ-S.

Serum *high-sensitivity C-reactive protein* (hs-CRP) was measured on Abbott ARCHITECT ci4100.

### Study endpoints

*The primary endpoint* was between-group difference of changes in hippocampal brain volumes from baseline to follow-up.

*Secondary exploratory endpoints* were between-group differences of changes in total and individual brain volumes, resting state functional connectivity, salivary cortisol levels, hs-CRP, and mental wellbeing questionnaires.

### Statistical analysis

Responses to interventions were evaluated as the change between baseline to post-intervention time points using a univariate ANOVA model corrected for age, sex, and baseline value. The least significant difference correction test was applied to each reported variable to test for between-group differences. False discovery rate correction was used to correct for all multiple comparisons, and two-sided *P* < .05 was considered statistically significant.

## Results

Of the 77 patients identified and 71 approached, 56 were randomized, with 52 completing the trial (age 66 ± 9 years, 91% women, 17 in the standard care arm, 16 in the physical exercise training arm, and 19 in the cognitive behavioural therapy arm). The groups were well balanced for age and sex and had a typical distribution of triggering factors, left ventricular ballooning phenotype, medical or mental health comorbidities, and medications at hospital discharge.

**Figure 1:**
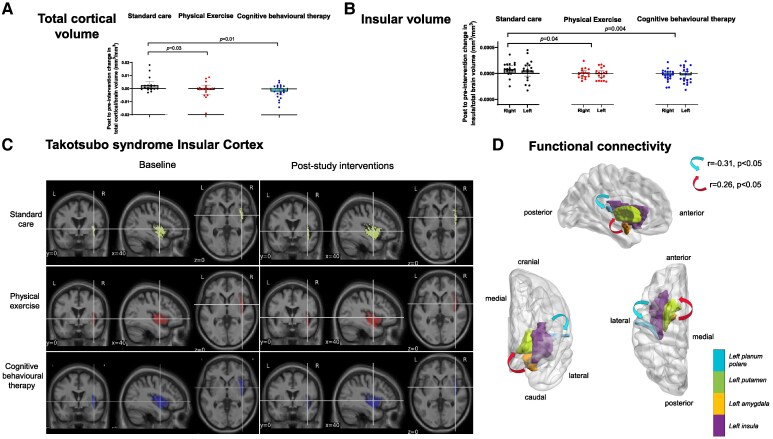
Post- to pre-intervention changes in total cortical volume (*A*) and right and left insula volumes (*B*) (all adjusted to total brain volume), for each study group, shown as individual data points with superimposed group mean and 95% confidence intervals. (*C*) Each group-averaged T1-weighted neuro-images showing baseline and post-intervention insular cortex volumes overlaid in yellow (standard care group), red (physical exercise group), and blue (cognitive behavioural therapy group). (*D*) Schematic representation of the maintained homeostatic functional connections in the physical exercise and cognitive behavioural therapy groups: sagittal (top), coronal (bottom, left), and axial (bottom, right) representations of the left brain hemisphere, showing the reduced functional connectivity (blue arrows) between the left insula and planum polare and increased functional connectivity (red arrows) between the left amygdala and the left putamen in the standard care arm

The primary outcome (hippocampal volume) was unaffected by exercise [7.3 × 10^−5^ (95% confidence interval −6.4 × 10^−5^ to 2.1 × 10^−4^)] or cognitive behavioural therapy [4.1 × 10^−5^ (−9.6 × 10^−5^ to 1.8 × 10^−4^)]. Physical exercise and cognitive behavioural therapy decreased total cortical volume [−4.6 × 10^−3^ (−8.6 × 10^−3^ to −5.2 × 10^−4^), *P* = .03 and −5.2 × 10^−3^ (−9.1 × 10^−3^ to −1.3 × 10^−3^), *P* = .01] and right insular volume [−8.3 × 10^−5^ (−1.7 × 10^−4^ to −1.4 × 10^−6^), *P* = .046 and −1.2 × 10^−4^ (−2.0 × 10^−4^ to −4.1 × 10^−5^), *P* = .004] in contrast to the standard care control arm (*Figure 1A–C*). Cognitive behavioural therapy resulted in an increase in the total cerebral white matter [5.7 × 10^−3^ (1.5 × 10^−3^ – 9.8 × 10^−3^), *P* = .009] and a reduction in total grey matter [−5.4 × 10^−3^ (−9.6 × 10^−3^ to −1.1 × 10^−3^), *P* = .01] relative to the standard care arm. There was no difference in white matter hyperintensities or structural connectivity between groups.

There were alterations in resting state functional connectivity in the standard care group compared with either physical exercise or cognitive behavioural therapy at two distinct levels: (i) reduced between the left insula and planum polare [*r* = −.31 (95% confidence interval, −.461 to −.159), *P* < .05)] and (ii) increased between the left amygdala and the left putamen [*r* = .26 (.131–.389), *P* < .05] (*Figure 1D*).

Both exercise and cognitive behavioural therapy decreased salivary cortisol stress responses at 45 min post-awakening [−52 (−84 to −21), *P* = .003 and −47 (−75 to −18), *P* = .004] compared with standard care.

There was a decrease in the Perceived Stress score [−5 (−10 to −1), *P* = .04] and hs-CRP [−1.7 (−3.4 to −.1), *P* = .038] with cognitive behavioural therapy compared with standard care.

Full recovery of the left ventricular ejection fraction, global longitudinal strain, and brain natriuretic peptide was observed in all three groups of patients.

## Discussion

We demonstrate that shortly after an acute episode of takotsubo cardiomyopathy, a 12-week behavioural modification intervention with either physical exercise training or cognitive behavioural therapy does not meet the primary endpoint of change in hippocampal volume, although it was associated with (i) improvements in total cortical and insular volumes, (ii) preservation of the homeostatic functional connections of both the insula and amygdala, and (iii) attenuation of systemic cortisol stress responses. Cognitive behavioural therapy, in particular, improves the patients’ perception of stress and results in reductions in systemic inflammation. These neurobiological improvements are uncoupled from the natural course of the spontaneous left ventricular ejection fraction recovery. Our preliminary observations reinforce the concept that behavioural modifications promote restoration of neurobiological resilience to stress,^9^ although we recognize that their frequency and intensity were higher than standard healthcare provision. The small sample size of a mechanistic study and relatively short follow-up may have not captured sustained structural and functional changes; therefore, larger and longer studies exploring clinical effectiveness of an integrated exercise and cognitive behavioural intervention are warranted.

## Declarations

### Data Availability

Anonymous participant data are available on reasonable request. These will be reviewed by the principal investigator and made available on assessing the merit of each request. Once such a request is approved, data can be made available on a secure online data sharing platform after signing of a data access agreement and confidentiality agreement.

### Pre-registered Clinical Trial Number

NCT05530135.
